# Diagnosis and Treatment of Angiography Positive Medium to Large Vessel Childhood Primary Angiitis of Central Nervous System (p-cPACNS): An International Survey

**DOI:** 10.3389/fped.2021.654537

**Published:** 2021-03-26

**Authors:** Angela S. Quan, Jürgen Brunner, Benjamin Rose, Martin Smitka, Gabriele Hahn, Clare E. Pain, Renate Häfner, Fabian Speth, Lucia Gerstl, Christian M. Hedrich

**Affiliations:** ^1^Department of Women's and Children's Health, Institute of Translational Medicine, University of Liverpool, Liverpool, United Kingdom; ^2^Pädiatrische Rheumatologie, Department Kinder- und Jugendheilkunde, Klinisches Ethikkomitee, Medizinische Universität Innsbruck, Innsbruck, Austria; ^3^Klinik und Poliklinik fur Kinder- und Jugendmedizin, Universitätsklinikum Carl Gustav Carus, Technische Universität Dresden, Dresden, Germany; ^4^Institut und Poliklinik für Radiologie, Universitätsklinikum Carl Gustav Carus, Technische Universität Dresden, Dresden, Germany; ^5^Department of Paediatric Rheumatology, Alder Hey Children's NHS Foundation Trust Hospital, Liverpool, United Kingdom; ^6^Deutsches Zentrum für Kinder- und Jugendrheumatologie, Garmisch-Partenkirchen, Germany; ^7^Zentrum für Geburtshilfe, Kinder- und Jugendmedizin, Klinik und Poliklinik für Kinder- und Jugendmedizin, Universitätsklinikum Eppendorf, Hamburg, Germany; ^8^Division of Paediatric Neurology, Developmental Medicine and Social Paediatrics, Department of Paediatrics, Dr. von Hauner Children's Hospital, Ludwig-Maximilians-University Munich, Munich, Germany

**Keywords:** vasculitis, CNS, childhood, pediatric, treatment, diagnosis, inflammation, CNS inflammation

## Abstract

Childhood Primary Angiitis of Central Nervous System (cPACNS) is rare, but can cause significant damage and result in disability or even death. Because of its rarity, the sometimes acute and variable presentation, limited awareness, and the absence of widely accepted diagnostic and therapeutic standards, cPACNS is a diagnostic and therapeutic challenge. Three subcategories of cPACNS exist, including angiography-positive non-progressive p-cPACNS, angiography-positive progressive p-cPACNS which both affects the medium to large vessels, and angiography-negative small vessel sv-cPACNS. Diagnosis and treatment of cPACNS relies on personal experience, expert opinion and case reports/case series. To collect information on diagnostic and therapeutic approaches to transient and progressive cPACNS, a survey was shared among international clinicians (German Society for Pediatric Rheumatology, the Pediatric Rheumatology European Society, the German speaking “Network Pediatric Stroke,” and members of the American College of Rheumatology/CARRA Pediatric Rheumatology list server). Results from this survey will be used to define statements toward a consensus process allowing harmonization of diagnostic and therapeutic approaches and the generation of evidence in a rare condition.

## Background

Childhood Primary Angiitis of Central Nervous System (cPACNS) is a rare, severe and potentially life threatening disease (1). While the pathophysiology of cPACNS remains largely unclear, seemingly untriggered inflammation results in immune cell infiltration and activation with subsequent destruction of arterial blood vessels. Vessel wall edema and thickening results in segmental stenosis, poor blood circulation and/or intracranial hemorrhage (2).

Due to the rarity of the condition and, variable clinical symptoms, cPACNS can be difficult to diagnose and manage. Presentation and prognosis are dependent on the size and location of affected vessels, and the severity and extent of the inflammatory response induced. The presence of (sometimes) age-specific differential diagnoses, including primary seizures, migraines, tumors, infections, etc., represent additional diagnostic challenges (3).

Though case definitions have been proposed, they vary between neurological and rheumatological/immunological literature, and no uniformly recognized guidelines for diagnosis and treatment exist (3, 4). Calabrese et al. (5, 6) proposed diagnostic criteria for adult-onset PACNS that were adjusted for childhood disease by Benseler et al. (7) (Table 1).

**Table 1:** Criteria for p-cPACNS.

**Pediatric criteria for p-PACNS (7–9)**
Newly acquired neurological deficit
Angiographic and/or histological features of angiitis within the CNS
No evidence of an underlying systemic disorder that explains the symptoms
Recently developed psychiatric deficits

Though helpful in some cases, criteria leave cPACNS a diagnosis of exclusion, and time to diagnosis and treatment are largely dependent on the awareness and experience of the treating team.

Childhood PACNS can be classified into three subcategories based on the size of vessels affected and disease course and progression (1). Angiography-positive p-cPACNS affects medium to large intracranial vessels, while angiography-negative disease affects the small sized vessels (sv-cPACNS). Within the group of p-cPACNS, non-progressive or transient disease usually unilaterally affects short segments of either *Arteria cerebri anterior* or *media*. While causing variable degree of damage during inflammatory activity, non-progressive p-cPACNS is self-limited within 3 months. Transient disease can be discriminated from progressive p-cPACNS that can affect either short or longer segments of one or more medium to large sized cerebral arteriae, including posterior vessels (1). In progressive p-cPACNS, the absence of sufficient treatment results in progressive narrowing of affected vessels on angiography after 3 or more months (10). Treatment of p-cPACNS is largely empiric and based on preliminary evidence (10), small case series and expert opinion (4).

To optimize and harmonize diagnostic and therapeutic approaches consensus treatment plans (CTP) can be a tool in rare conditions where clinical trials are currently not available or realistic, such as p-cPACNS. They allow prospective data collection on therapy and associated outcomes. As a first step, real-life standard of care requires to be documented to develop statements e.g., through Delphi surveys that can then be used toward an expert consensus conference for CTP development (11, 12).

We describe a survey undertaken with international experts in which we sought information on diagnostic and therapeutic strategies in transient and progressive p-cPACNS.

## Methods

### Instrument

A survey was designed to collect information on diagnostic and therapeutic approaches in p-cPACNS by experts in the field. It consisted of introductory questions addressing demographics (sub-specialty, country of practice) and experience of participants, and 2 case scenarios accompanied by multiple choice questions. One case (case 1) represented a patient with progressive p-cPACNS, the second (case 2) had two alternative outcomes: transient p-cPACNS likely not related to VZV, and transient p-cPACNS likely related to VZV. Survey details can be accessed in Supplementary Material 1. Case based questions aimed at determining examinations deemed important to diagnose p-cPACNS. The survey also queried how participants would treat and follow patients with transient or progressive pPACNS. Lastly, respondents were asked which specialties they consider important to be involved in the diagnosis and treatment of p-cPACNS patients. Multiple choice answers were provided, as well as the option to add comments and/or additional answers (Supplementary Material 1).

The survey was conducted online using the web-based tool Survey Monkey (Survey Monkey Inc.; California, USA; www.surveymonkey.com). A link to the survey was sent to addressees in August 2019 and the survey was open for 2 months. A reminder e-mails was sent out at both 4 and 6 weeks after the initial survey link was shared. The survey was distributed among colleagues from Europe, North America, and globally with experience in the diagnosis and treatment of p-cPACNS. This was achieved through member email lists of the German Society for Pediatric Rheumatology (GKJR) (*n* = 151; Pediatric Rheumatologists; personal email), the Pediatric Rheumatology European Society (PRES) (*n* = 7,800; society members; monthly PRES email newsletter), members of the German speaking “Network Pediatric Stroke” with members in Germany, Austria and Switzerland (*n* = 72; including Pediatric Rheumatologists, Immunologists, Neurologists and specialists for pediatric haemostaseology; personal email), and subscribers to the American College of Rheumatology/CARRA Pediatric Rheumatology Bulletin Board (ped-rhe-list-bounces@mcmaster.ca) (*n* = 1849; personal email).

### Analysis of Response

Descriptive analysis of responses was performed using Microsoft Excel (Redmond, Washington, USA).

## Results

### Responses, Demographics and Experience of Participants

**Figure 1:**
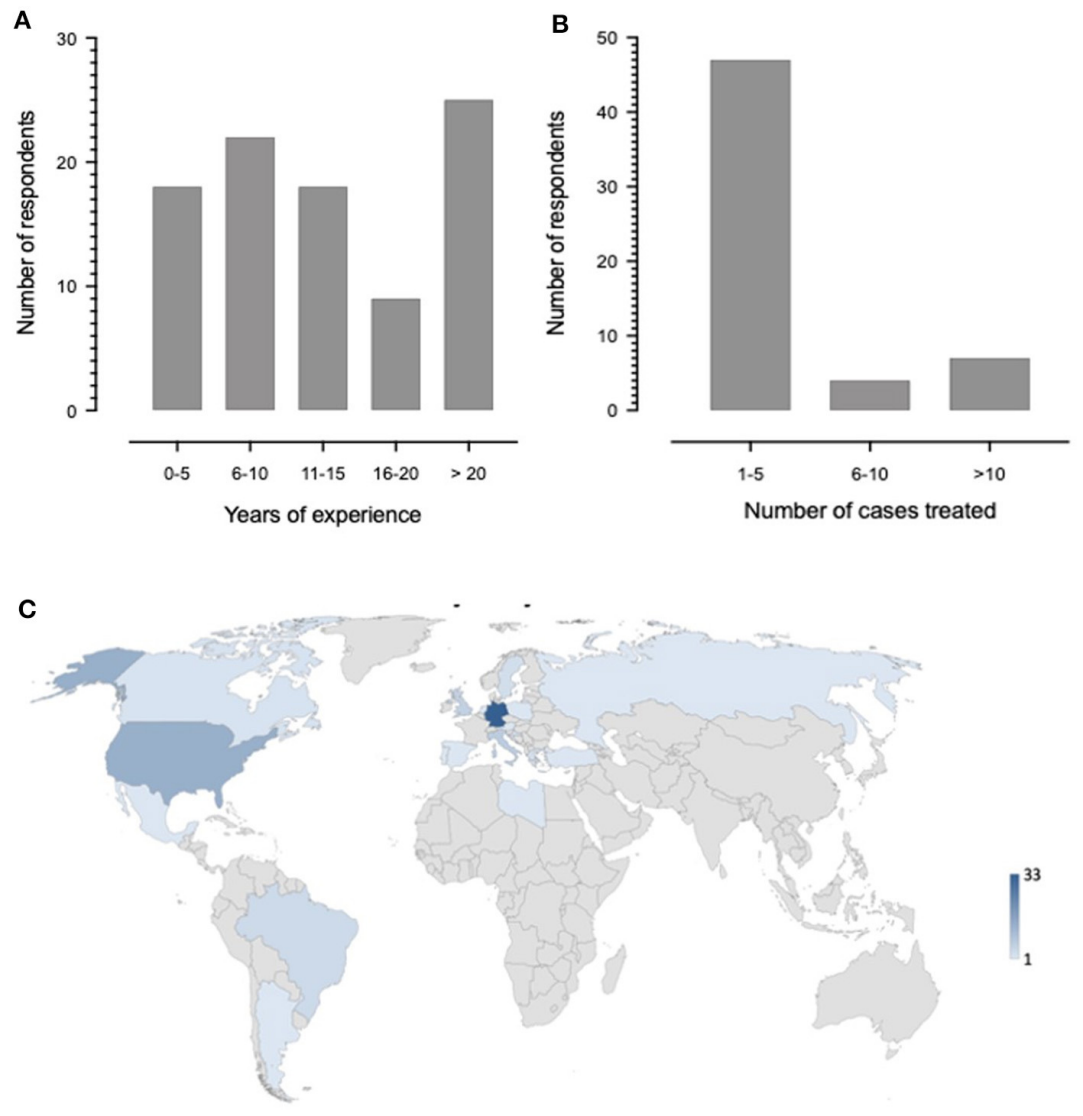
Demographics of participants. **(A)** Number of years of experience clinicians have working in their subspecialty. **(B)** Number of patients with p-cPACNS respondents have treated. One of the responders answered to having treated more than 100 cases. **(C)** Map depicting which countries the respondents work in. The number of respondents from the countries are shown in the legend using a shading scale. The darker shading indicates more responses were from those countries.

The survey was answered by a total of 92 clinicians, with the majority of colleagues specializing in Pediatric Rheumatology (72, 78.2%), followed by 11 Pediatric Neurologists (11.9%), 4 General Pediatricians (4.3%), one Adult Rheumatologist, Pediatric Intensive Care Clinician, Pediatric Hematologist and Adult Neurologist each (Q1). The number of responses varied slightly between individual questions; the number of responses to each question are indicated throughout this manuscript. Experience of responders in their subspecialty varied, as did number of cases of cPACNS treated (Figures 1A,B, Q2, *N* = 92 responses). Figure 1C shows the distribution of where responders practiced (Q3, *N* = 85 responses).

### Diagnostic Approach

**Figure 2:**
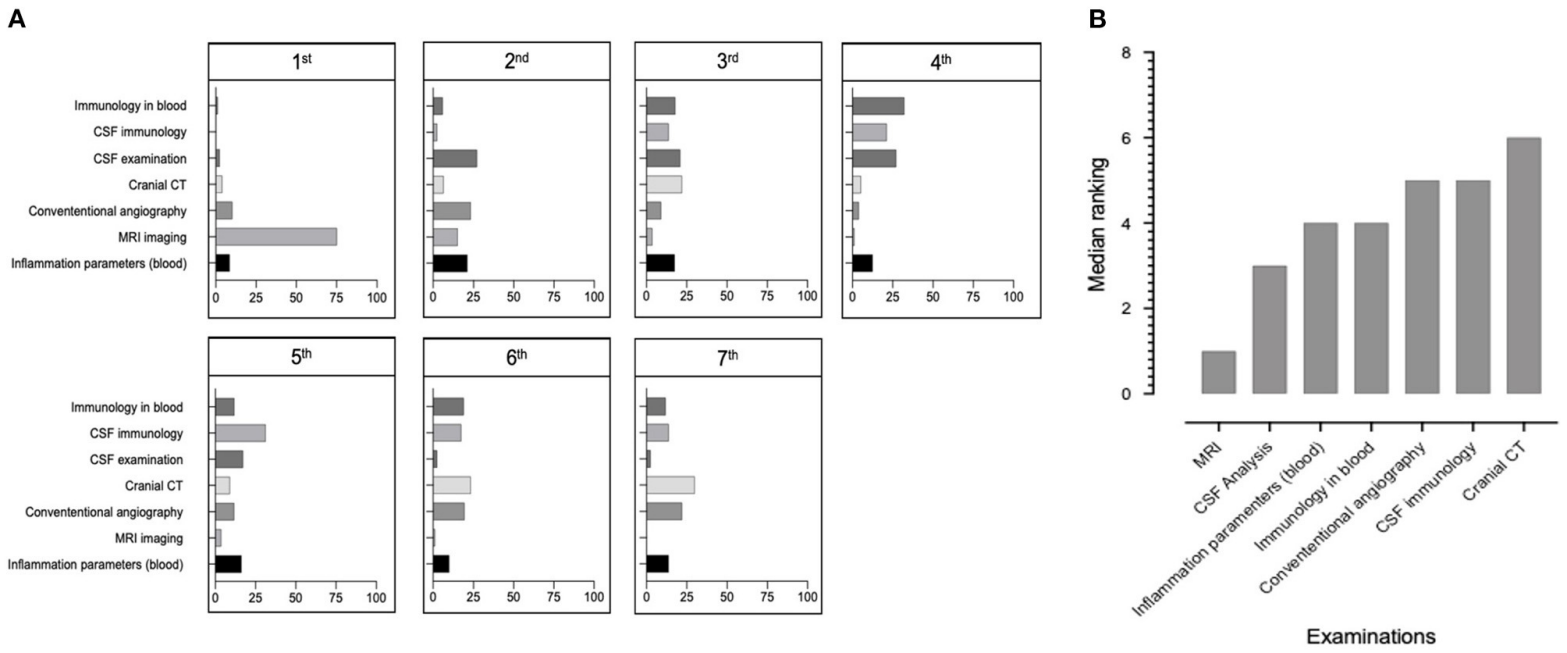
Importance of diagnostic tools. **(A)** Bar chart data illustrating the percentage of respondents who indicated which diagnostic tests they thought was more important. Respondents were asked to rank the following examinations according to their importance for the diagnosis of p-cPACNS from 1 being the most important and 7 being the least important (*n* = 86). **(B)** Bar chart using median data illustrating which diagnostic tests respondents ranked as most important (1) and least importance (7). (*n* = 86).

To understand how participants approach the diagnosis of p-cPACNS, they were asked to rank 7 clinical, laboratory and imaging examinations weighing their importance for the diagnosis of pPACNS (Q5, *N* = 86 responses, Figure 2A). Median importance ranks (1–7) assigned to individual examinations were calculated, where 1 indicated the most important (Figure 2B). MRI imaging was most commonly ranked as the most important examination (median: 1), followed by CSF analysis (median: 3), inflammation parameters in blood, immunology in the blood (ANA, ENA, ds-DNA, ANCA, antiphospholipid antibodies, rheumatoid factor, complement system components and activation) (median: 4 both), conventional angiography, CSF immunology (encephalitis-associated antibodies) (median: 5 both) and lastly cranial CT (median: 6).

When asked whether genetic testing should be considered, 50/87 respondents (57.5%) felt that genetic testing should indeed be performed (Q6, *N* = 87 responses), and among 34 respondents who specified, most suggested testing for deficiency of adenosine deaminase 2 (DADA2; 21/34; 61.8%). Further conditions mentioned included Ehlers-Danlos and Marfan syndromes (2.9% each), systemic and organ-specific inflammatory disorders, such as granulomatosis (familial), hemophagocytic lymphohistiocytosis (HLH), Systemic lupus erythematosus (SLE), Sjögren's syndrome, collagen tissue disease, and Moya Moya disease.

### Case Studies

After aforementioned general questions, the survey presented two clinical scenarios addressing specific diagnostic and therapeutic approaches.

#### Case 1

An 8-year-old boy presents with increasing fatigue (past 2 weeks) and new acute-onset symptoms of aphasia, ataxia, headaches, and progressive vertigo for the past 24 h. He has no personal of family history of clotting disorders, strokes or autoimmune/inflammatory disease. He has had no infections in the past year and no travel history (other than a “sore throat or sniffles here and there”), no pets, and no other symptoms.

When asked about diagnostic approaches to this specific case (Q7, *N* = 74 responses), respondents ranked emergency MRI of the brain including angio-MRI (90.5%) and blood tests including full blood counts, inflammatory markers and clotting tests (90.5%) as most important. These were followed by lumbar puncture to analyze the CSF (82.4%), and brain CT scan including CT angiography (23.0%). Of the respondents who considered blood tests essential (Q8, *N* = 74 responses), 98.6% would order full blood count including complete white cell count, 97.3% would order clotting tests including PTT, INR, fibrinogen and D dimers, and 94.6% would order immunology tests including ANA, ENA, complement factors, cardiolipin antibodies and ANCA. When asked which blood immunology tests they would order, 97.3% answered anti-phospholipid antibody testing, followed by antinuclear antibody testing (93.2%), requested anti-neutrophil cytoplasmic antibodies (89.2%), anti-dsDNA antibodies (87.8%), complement factors and activation of the complement cascade (83.8%), and anti-NMDA (N-methyl-D-aspartate) receptor and aquaporin antibodies (67.6%) (Q9, *N* = 74 responses). Of 74 respondents who considered performing a lumbar puncture (Q10, *N* = 74 responses), most (98.6%) would request cell counts, differentiation and protein quantification, followed by microbial culture (93.2%), glucose levels (90.5%), oligoclonal bands (89.1%), CSF opening pressure (86.5%), lactate (74.3%) and anti-NDMA and aquaporin antibodies (68.9%). When asked about helpful MRI techniques (Q11, *N* = 71 responses), 87.3% of responders requested MRI angiography, followed by fluid-attenuated inversion recovery (FLAIR; 55/71; 77.5%), diffusion-weighted MRI sequences (53/71, 74.7%), T1 with fat saturation (FS; 30/71; 42.3%), T1FS with contrast medium (38/71, 53.5%), T2FS (37/71, 52.1%), Turbo inversion recovery magnitude (TIRM)/Short tau inversion recovery (STIR; 28/71; 39.4%). Detailed results are summarized in Table 2.

**Table 2:** Diagnostic approach in case 1.

**Diagnostic approach (Q7)**			
1	MRI of the brain including angio-MRI	67/74	90.5%
1	Blood tests including full blood counts, inflammatory markers and clotting tests	67/74	90.5%
3	Lumbar puncture to analyse the CSF	61/74	82.4%
4	Brain CT scan including CT angiography	17/74	23.0%
**Blood tests deemed essential (Q8)**			
1	Complete white cell count	73/74	98.6%
2	Clotting tests including PTT, INR, fibrinogen and D dimers	72/74	97.3%
3	Immunology tests including ANA, ENA, complement factors, cardiolipin antibodies and ANCA	70/74	94.6%
4	Interferon gamma release assay (IGRA) for Tuberculosis	38/74	51.3%
	Adenosine deaminase (ADA) 2 activity	38/74	51.3%
**Blood immunology tests (Q9)**			
1	Anti-phospholipid antibody testing	69/74	97.3%
2	Antinuclear antibodies	69/74	93.2%
3	Anti-neutrophil cytoplasmic antibodies	66/74	89.2%
4	Anti-dsDNA antibodies	65/74	87.8%
5	Complement factors and activation of the complement cascade	62/74	83.8%
6	Anti-NMDA (N-methyl-D-aspartate) receptor and aquaporin antibodies	50/74	67.6%
7	No blood immunology tests are necessary	2/74	2.7%
8	“Others,” including: aquaporin antibodies depending on the MRI image, Anti-myelin oligodendrocyte glycoprotein (MOG) antibodies and Mayo clinic encephalitis panel	3/74	4.1%
**Lumbar puncture (Q10)**			
1	Cell counts, differentiation	73/74	98.6%
1	Protein quantification	73/74	98.6%
2	Microbial culture	69/74	93.2%
3	Glucose	67/74	90.5%
4	Oligoclonal bands	66/74	89.1%
5	CSF opening pressure	64/74	86.5%
6	Lactate	55/74	74.3%
7	Anti-NDMA and aquaporin antibodies	51/74	68.9%
**MRI techniques (Q11)**			
1	MRI angiography	62/71	87.3%
2	Fluid-attenuated inversion recovery (FLAIR)	55/71	77.5%
3	Diffusion-weighted MRI sequences	53/71	74.7%
4	T1 with fat saturation (FS)	30/71	42.3%
5	T1FS with contrast medium	38/71	53.5%
6	T2FS	37/71	52.1%
7	Turbo inversion recovery magnitude (TIRM)/Short tau inversion recovery (STIR)	28/71	39.4%

Next, respondents were informed that the patient exhibited elevated ESR (30mm/h) and CRP (4mg/L). Approximately 30 h after the onset of ataxia and aphasia, Magnetic Resonance Imaging (MRI) shows alterations in proton diffusion capacity in the Cerebellum and a significant and long ranging stenosis of the distal Basilar artery in MRI angiography (Supplementary Material 1).

Based on new information provided, respondents were asked to select 5 most likely differential diagnoses (Q12, *N* = 73 responses). Most respondents selected progressive p-cPACNS (80.8%), followed by transient p-cPACNS (69.9%), ischaemic stoke (64.4%), congenital anatomical deformity (39.7%) and infections (38.4%). Asking indicators of likely progressive p-cPACNS (Q13, *N* = 73 responses), the majority of respondents suggested imaging results (87.7%), followed by clinical course with disease progression over 3 months or more (69.9%), laboratory findings suggesting systemic inflammation (46.6%), acute presentation with “systemic signs” (43.8%), and response to immune modulation (41.1%). Detailed results are summarized in Table 3.

**Table 3:** Most likely diagnoses in case 1.

**5 most likely differential diagnoses (Q12)**			
1	Selected progressive p-cpacns	59/73	80.8%
2	Transient *p-cpacns*	51/73	69.9%
3	Ischaemic stoke	47/73	64.4%
4	Congenital anatomical deformity	29/73	39.7%
5	Infections	28/73	38.4%
6	Arteriovenous (AV) malformation	26/73	35.6%
7	CNS tuberculosis	24/73	32.9%
8	Migraine	11/73	15.1%
8	Tumo r	11/73	15.1%
10	Multiple sclerosis	4/73	5.5%
11	Traumatic intracranial bleeding	3/73	4.1%
**Indicators of likely progressive p-cPACNS (Q13)**			
1	Imaging results, namely involvement of more than one vessel, involvement of distal segments, and posterior vessel affected	64/73	87.7%
2	Clinical course with disease progression over 3 months or more	51/73	69.9%
3	Laboratory findings suggesting systemic inflammation	34/73	46.6%
4	Acute presentation with “systemic signs”	32/73	43.8%
5	Response to immune modulation	30/73	41.1%

Next, the following information was provided: Autoantibodies in CSF and blood come back negative, there's no evidence for clotting disorders or infection, including TB. Systemic inflammatory parameters remain normal. Based on the involvement of posterior arteries, the diagnosis of (likely) progressive p-cPACNS (Childhood Primary Angiitis of Central Nervous System) is made.

Participants were asked to decide which medication they consider for the induction of remission (Q14, *N* = 73 responses). Sixty-eight of 73 respondents would start treatment with intravenous Methylprednisolone (IVMP) over 5 days, followed by oral prednisolone (93.2%), 47/73 (64.3%) consider treatment with intravenous Cyclophosphamide every month for 4–6 months, 9/73 (12.3%) would start treatment with mycophenolate mofetil (MMF), and 3/73 (4.1%) would treat with oral prednisolone. None of the respondents chose oral cyclophosphamide or azathioprine for induction treatment.

Considering prophylaxis of thromboembolic events (Q15, *N* = 70 responses), most responders agreed on administering IV heparin initially (65.7%), followed by acetyl salicylic acid (ASA), and a combination of ASA and clopidogrel. Considering post-acute phase thrombosis prophylaxis (Q16, *N* = 70 responses), the majority of respondents would prescribe aspirin (47.1%), followed by subcutaneous heparin (22.9%), a combination of ASA and clopidogrel (14.3%), warfarin (11.4%), clopidogrel alone (5.7%), or DOACs (2.9%). Opinions on when to discontinue anticoagulation treatments (Q20, *N* = 70 responses) were divided and ranged between 3 and 36 months. One respondent would not administer anticoagulation treatment (1.4%) (Table 4).

**Table 4:** Treatment decisions in case 1.

**Induction of remission (Q14)**			
1	Intravenous Methylprednisolone (IVMP) over 5 days, followed by oral prednisolone	68/73	93.2%
2	Intravenous Cyclophosphamide every month for 4-6 months	47/73	64.3%
3	Mycophenolate mofetil (MMF)	9/73	12.3%
4	Oral prednisolone	3/73	4.1%
**Prophylaxis of thromboembolic events (Q15)**			
1	IV heparin initially	46/70	65.7%
2	Acetyl salicylic acid (ASA)	19/70	27.1%
3	Combination of ASA and clopidogrel	5/70	7.1%
4	“Others”: including low molecular weight heparin, Heparin 100 IU/kg/12hr	13/70	18.6%
-	Warfarin, clopidogrel alone or direct oral anticoagulants (DOAC);	0	0
**Post-acute phase thrombosis prophylaxis (Q16)**			
1	ASA	33/70	47.1%
2	Subcutaneous heparin	16/70	22.9%
3	Combination of ASA and clopidogrel	10/70	14.3%
4	Warfarin	8/70	11.4%
5	Clopidogrel	4/70	5.7%
6	DOACs	2/70	2.9%
7	None	1/70	1.4%
**Duration of thrombosis prophylaxis (Q17)**			
	3 months	7/70	10%
	6 months	9/70	12.9%
	12 months	13/70	18.6%
	18 months	3/70	4.3%
	24 months	13/70	18.6%
	36 months	9/70	12.9%
	None	1/70	1.4%
**Immune modulating maintenance treatment (Q18)**			
1	MMF	53/73	72.6%
2	Azathioprine	15/73	20.5%
3	Oral prednisolone	13/73	17.8%
4	Oral cyclophosphamide (following *Fauci* scheme)	1/73	1.4%
4	TNF inhibitors	1/73	1.4%
**Duration of immune modulating treatment (Q19)**			
	3 months	3/73	4.1%
	6 months	4/73	5.5%
	12 months	12/73	16.4%
	18 months	14/73	19.2%
	24 months	27/73	37.0%
	36 months	4/73	5.5%
**Duration of oral corticosteroid treatment, including slow taper (Q20)**			
	3 months	15/72	20.8%
	6 months	30/72	41.7%
	12 months	11/72	15.3%
	18 months	3/72	4.2%
	24 months	4/72	5.6%
	36 months	1/72	1.4%
	None	1/72	1.4%

Considering immune modulating maintenance treatment (Q17, *N* = 73 responses), the majority of respondents (72.6%) would prescribe MMF, followed by azathioprine (20.5%), oral prednisolone (17.8%), intravenous cyclophosphamide (13.7%), rituximab (9.6%), methotrexate (5.5%), oral cyclophosphamide (following *Fauci* scheme) or TNF inhibitors (1.4% each). Opinions on the required duration of treatment were divided (Q18, *N* = 73 responses) and ranged between 3 and 36 months. When asked how long to include oral corticosteroid treatment in the regimen, including slow taper (Q19, *N* = 72 responses), respondents responded: 3 months (20.8%), 6 months (41.7%), 12 months (15.3%), 18 months (4.2%), 24 months (5.6%), 36 months (1.4%), and 1.4% indicated no corticosteroid treatment (Table 4).

Monitoring disease activity and damage using MRI (Q21, *N* = 72 responses), 56/72 (77.8%) respondents suggested to repeat MRI after 3 months, 36/72 after 6 months (50.0%), 25/72 after 12 months (34.7%), 11/72 after 18 months (15.3%), 21/72 after 24 months (29.1%), and 10/72 after 36 months (13.9%) and 11/72 answered other (15.3%). When asked when to schedule clinical follow up (Q22, *N* = 73 responses), 59/73 respondents indicated within 3 months (80.8%), 23/73 after 6 months (31.5%), 22/73 after 12 months (30.1%), 19/73 after 18 months (26.0%), 21/73 after 24 months (28.8%) and 15/73 after 36 months (20.6%).

#### Case 2

A 4-year-old girl presents with headaches and symptoms suggestive of a cerebrovascular stroke (vomiting with some language and speech delays). She has a past medical history of a clinically diagnosed Varicella Zoster Virus (VZV) infection 6 months ago. There's no history of strokes or clotting disorders in her personal or family history.

Similarly to case 1 (Q24, *N* = 70 responses), the majority of respondents suggested that initial investigations should include blood test (94.3%), emergency MRI of the brain including an MRI angiography (92.9%), lumbar puncture and CSF analysis (88.6%), and brain CT scan including CT angiography (31.4%). Of the participants who considered blood tests essential (Q25, *N* = 70 responses), 98.6% would request full blood count including complete white cell count; 97.1% clotting tests including PTT, INR, fibrinogen and D dimers, and 90.0% would order immunology tests including ANA, ENA, complement factors, cardiolipin antibodies and ANCA; 42.9% suggested blood tests for adenosine deaminase 2 activity (ADA2) and 41.4% for interferon gamma release assay (IGRA) to exclude tuberculosis (Table 5).

**Table 5:** Diagnostic approach in case 2.

**Diagnostic approach (Q24)**			
1	Blood tests including full blood counts, inflammatory markers and clotting tests	66/70	94.3%
1	Emergency MRI of the brain including an MRI angiography	65/70	92.9%
3	LUMBAR puncture to analyze the CSF	62/70	88.6%
4	Brain CT scan including CT angiography	22/70	31.4%
**Blood tests deemed essential (Q25)**			
1	Complete blood cell count, including differential blood count	69/70	98.6%
2	Clotting tests including PTT, INR, fibrinogen and D dimers	68/70	97.1%
3	Immunology tests including ANA, ENA, complement factors, cardiolipin antibodies and ANCA	63/70	90.0%
4	Adenosine deaminase (ADA) 2 activity	30/70	42.9%
5	Interferon gamma release assay (IGRA) to exclude tuberculosis	29/70	41.4%
**Blood immunology tests (Q26)**			
1	Anti-phospholipid antibody testing	66/70	94.3%
2	Antinuclear antibodies	63/70	90.0%
3	Anti-neutrophil cytoplasmic antibodies (ANCA)	64/70	91.4%
4	Anti-dsDNA antibodies	57/70	81.4%
5	Complement factors and activation of the complement cascade	53/70	75.7%
6	Anti-NMDA (N-methyl-D-aspartate) receptor and aquaporin antibodies	38/70	54.3%
**Lumbar puncture (Q27)**			
1	Cell counts, differentiation and protein quantification	69/70	98.6%
2	Protein	67/70	95.7%
3	Glucose	65/70	92.9%
4	Microbial cultures	63/70	90.0%
5	Opening pressure	59/70	84.3%
6	Oligoclonal bands	57/70	81.4%
7	Lactate	55/70	78.5%
8	Anti-NMDA and aquaporin antibodies	38/70	54.3%
**MRI techniques (Q28)**			
1	MRI angiography	60/68	88.2%
2	Fluid-attenuated inversion recovery (FLAIR)	50/68	73.5%
3	Diffusion-weighted MRI sequences	50/68	73.5%
4	T1 with fat saturation (FS)	37/68	54.4%
5	T2FS	34/68	50.0%
6	T1FS with contrast medium	28/68	41.2%
7	TIRM/STIR	27/68	39.7%

When asked about blood immunology (Q26, *N* = 70 responses), 94.3% considered anti-phospholipid antibodies as required, followed by antinuclear antibodies, ANCA, anti-dsDNA antibodies, complement factors and activation of the complement cascade, and anti-NMDA and aquaporin antibodies. Of the 70 participants requesting a lumbar puncture (Q27, *N* = 70 responses), 98.6% considered cell counts and differentiation, followed by protein (95.7%), glucose (92.9%), microbial cultures (90.0%), opening pressure (84.3%), oligoclonal bands (81.4%), lactate (78.5%), and anti-NMDA and aquaporin antibodies (54.3%). Of 68 participants who considered emergency MRI (Q28, *N* = 68 responses), 88.2% requested MRI angiography, followed by FLAIR (73.5%), diffusion-weighted sequences (73.5%), T1FS with contrast medium (54.4%), T2FS (50.0%), T1 with FS (41.2%), and TIRM/STIR (39.7%) (Table 5).

Next, the participants were informed that autoantibodies in CSF and blood come back negative, there's no evidence for clotting disorders or infection (including negative for TB and VZV PCR in CSF, serum VZV IgG positive, IgM borderline positive). Blood and CSF inflammatory markers remain within normal limits. DWI sequences unveiled altered diffusion capacity in the left hemisphere; Time of flight MR Angiography (TOF-MRA-)sequences demonstrate narrow caliber of left distal internal carotid artery and proximal anterior and medial cerebral artery. Post-gadolinium MRI sequences reveal contrast enhancement of the thickened vascular wall in the affected segments. Conventional angiography showed incomplete occlusion of the left A. cerebri media (Supplementary Material 1).

Respondents were then asked to choose the 5 most important differential diagnoses to consider (Q29, *N* = 70 responses). Most respondents (81.4%) considered p-cPACNS, likely transient related to VZV, followed by ischaemic stroke (70.0%), transient p-cPACNS not related to VZV (62.9%), CNS tuberculosis (61.4%) and infections e.g., meningitis (32.9%) (Table 6).

**Table 6:** Most likely diagnoses in case 2.

**5 most likely differential diagnoses (Q12)**			
1	p-cPACNS, likely transient related to VZV	57/70	81.4%
2	Ischaemic stroke	49/70	70.0%
3	Transient p-cPACNS not related to VZV	44/70	62.9%
4	CNS tuberculosis	43/70	61.4%
5	Infections e.g., meningitis	23/70	32.9%

When asked which medication to administer for induction treatment (Q30, *N* = 69 responses), 53/69 (76.8%) respondents suggested IVMP over 5 days, followed by 18/69 who considered intravenous Cyclophosphamide every month for 4–6 months (26.1%), 12/69 MMF (17.4%), 9/69 (13.0%) oral Prednisolone, and 2/69 Azathioprine (2.9%). None of the participants chose oral Cyclophosphamide for induction treatment. Respondents were asked how long to continue oral corticosteroid treatment including slow taper for (Q34, *N* = 66 responses), they answered: 3 months (25/66, 37.9%), 6 months (23/66, 34.9%), 12 months (7/66, 10.6%), 18 months (0/66, 0.0%), 24 months (2/66, 3.0%) and 36 months (1/66, 1.5%). Five of 66 respondents answered no to prescribing oral corticosteroid treatment (7.6%).

Considering prophylaxis of thromboembolic events (Q31, *N* = 67 responses), most responders agreed on administering IV Heparin initially as anticoagulation treatment (52.2%) in the acute phase, followed by ASA (29.9%), a combination of ASA and clopidogrel (6.0%) and warfarin (4.5%). Considering post-acute phase prophylaxis (Q32, *N* = 66 responses), a majority of responders would prescribe Aspirin (53.0%), followed by subcutaneous Heparin (18.2%), a combination of Aspirin and Clopidogrel (9.1%), warfarin (7.6%), clopidogrel or DOACs (4.6% each). Regarding discontinuation of anticoagulation treatments (Q35, *N* = 63 responses), respondents answered after 3 months (11.1%), 6 months (17.5%), 12 months (20.6%), 18 months (3.2%), 24 months (12.7%) and 35 months (6.4%). Five of 63 respondents answered they would not administer anticoagulation treatment (7.9%) (Table 7).

**Table 7:** Anticoagulation in case 2.

**Prophylaxis of thromboembolic events (Q31)**			
1	IV heparin initially	35/67	52.2%
2	Acetyl salicylic acid (ASA)	20/67	29.9%
3	Combination of ASA and clopidogrel	4/67	6.0%
4	Warfarin	3/67	4.5%
-	Clopidogrel alone or direct oral anticoagulants (DOAC);	0	0
**Post-acute phase thrombosis prophylaxis (Q32)**			
1	ASA	35/66	53.0%
2	Subcutaneous heparin	12/66	18.2%
3	None	8/66	12.1%
4	Combination of ASA and clopidogrel	6/66	9.1%
5	Warfarin	5/66	7.6%
6	Clopidogrel	3/66	4.6%
7	DOACs	3/66	4.6%
**Duration of thrombosis prophylaxis (Q35)**			
	3 months	7/63	11.1%
	6 months	11/63	17.5%
	12 months	13/63	20.6%
	18 months	2/63	12.7%
	24 months	8/63	18.6%
	36 months	4/63	6.4%
	None	5/63	7.9%

Monitoring of disease activity and damage using MRI (Q36, *N* = 69 responses) was suggested after 3 months by 60/69 (87.0%) respondents, after 6 months by 32/69 (46.4%) and after 12 months by 25/69 (36.2%). Eight of 69 respondents would repeat MRI after 18 months (11.6%), 17/69 after 24 months (24.6%) and 6/69 after 36 months (8.7%). Clinical follow up (Q37, *N* = 70 responses) was considered reasonable at 3 months by 58/70 respondents (82.9%), at 6 months by 24/70 (34.3%), 12 months by 24/70 (34.3%), 18 months by 16/70 (22.9%), 24 months by 18/70 (25.7%), and after 36 months by 14/70 (20.0%).

To assess how participants would alter their approach based on evidence of VZV infections temporally associated with p-cPACNS, they were provided with alternative test results: After the first line investigations, suppose the autoantibodies in CSF and blood come back negative, there's no evidence for clotting disorders, but the VZV PCR in the CSF comes back as positive.

As a result of positive testing for VZV (Q38, *N* = 68 responses), the majority of the respondents indicated they would treat the patients with IV Acyclovir treatment over 14 days (58/68, 85.3%), 32/68 (47.1%) of responders indicated initially treating with IVMP over 5 days followed by oral Prednisolone. Less common answers indicated by participants were induction with oral Prednisolone, followed by oral Prednisolone taper (10/68, 14.7%), IV Cyclophosphamide (2/68, 2.9%), MMF induction treatment (2/68, 2.9%), Azathioprine (1/68, 1.5%). None of the participants indicated they would treat the patient with oral Cyclophosphamide (*Fauci* scheme).

### Medical Specialties Involved

**Figure 3:**
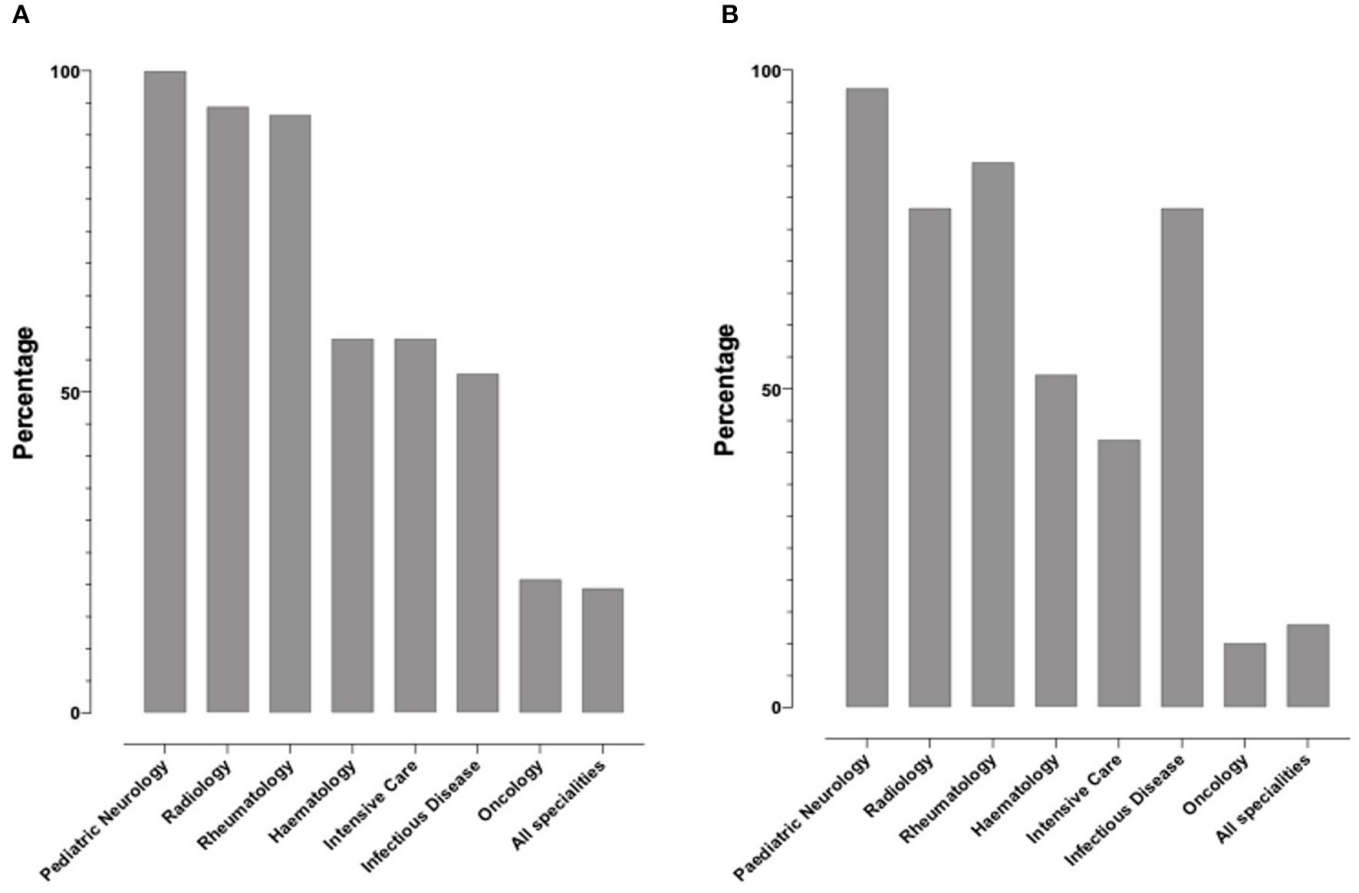
Percentage of specialties respondents indicated should involve in the treatment of the two cases. **(A)** Bar chart illustrating the percentage of respondents who have indicated which specialties should be involved in Case 1, **(B)** Respondents were also asked to indicated which specialties should be involved in treating Case 2.

For both cases, participants were asked to indicate which specialties should be involved in the patient's care (Figure 3A, Q23, *N* = 72 responses and Figure 3B, Q39, *N* = 69 responses). Answers suggested multi-professional approaches in both clinical scenarios.

### Influence of Experience in Approach to Diagnosis and Treatment

**Figure 4:**
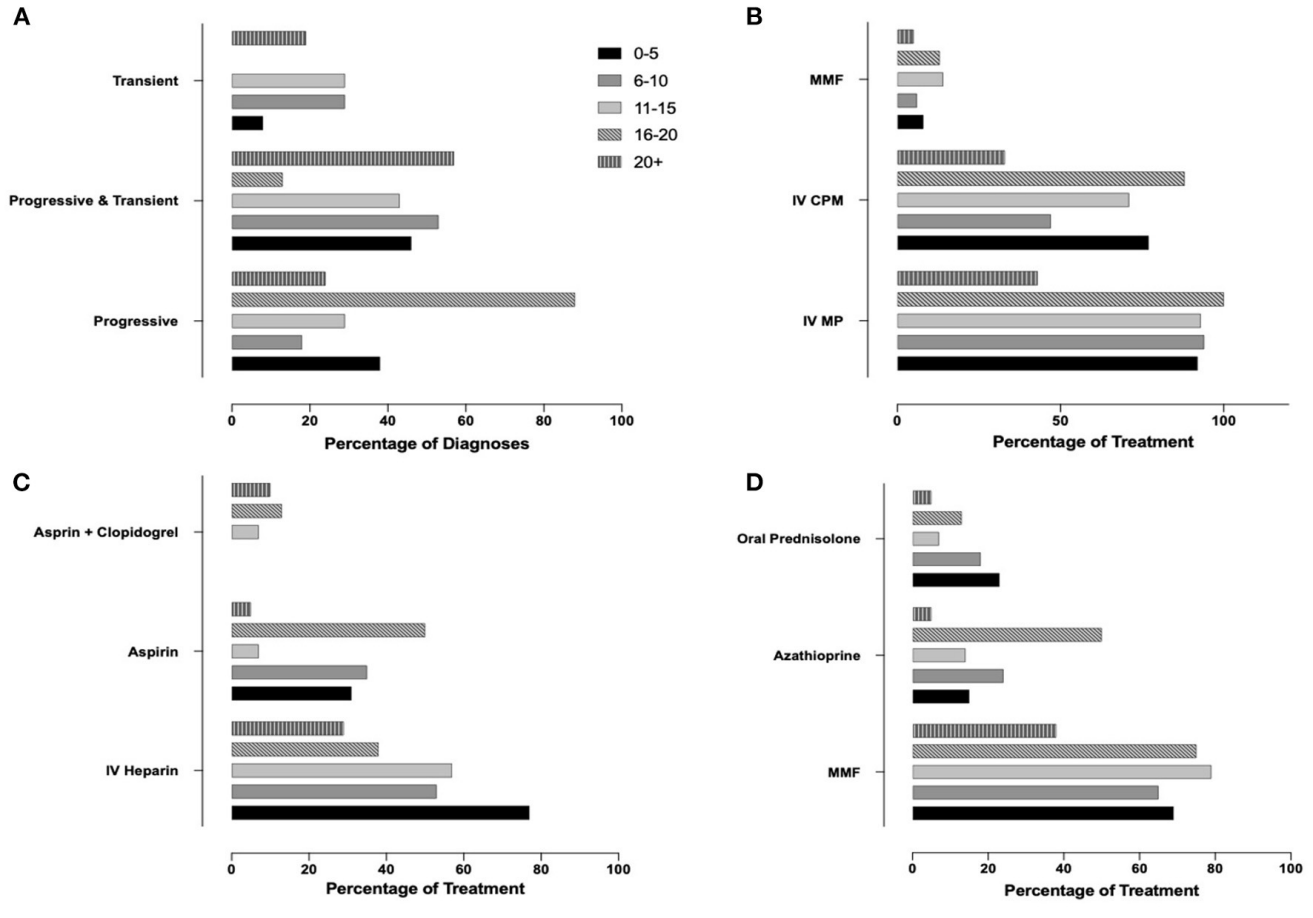
Influence of Experience in Approach to Diagnosis and Treatment in case 1. **(A)** Correlation between number of years of experience clinicians had and their diagnosis of the patient in case 1. **(B)** Years of experiences vs. Induction treatment. **(C)** Years of experience vs. Acute anticoagulation treatment. **(D)** Years of experience vs. Maintenance treatment.

**Figure 5:**
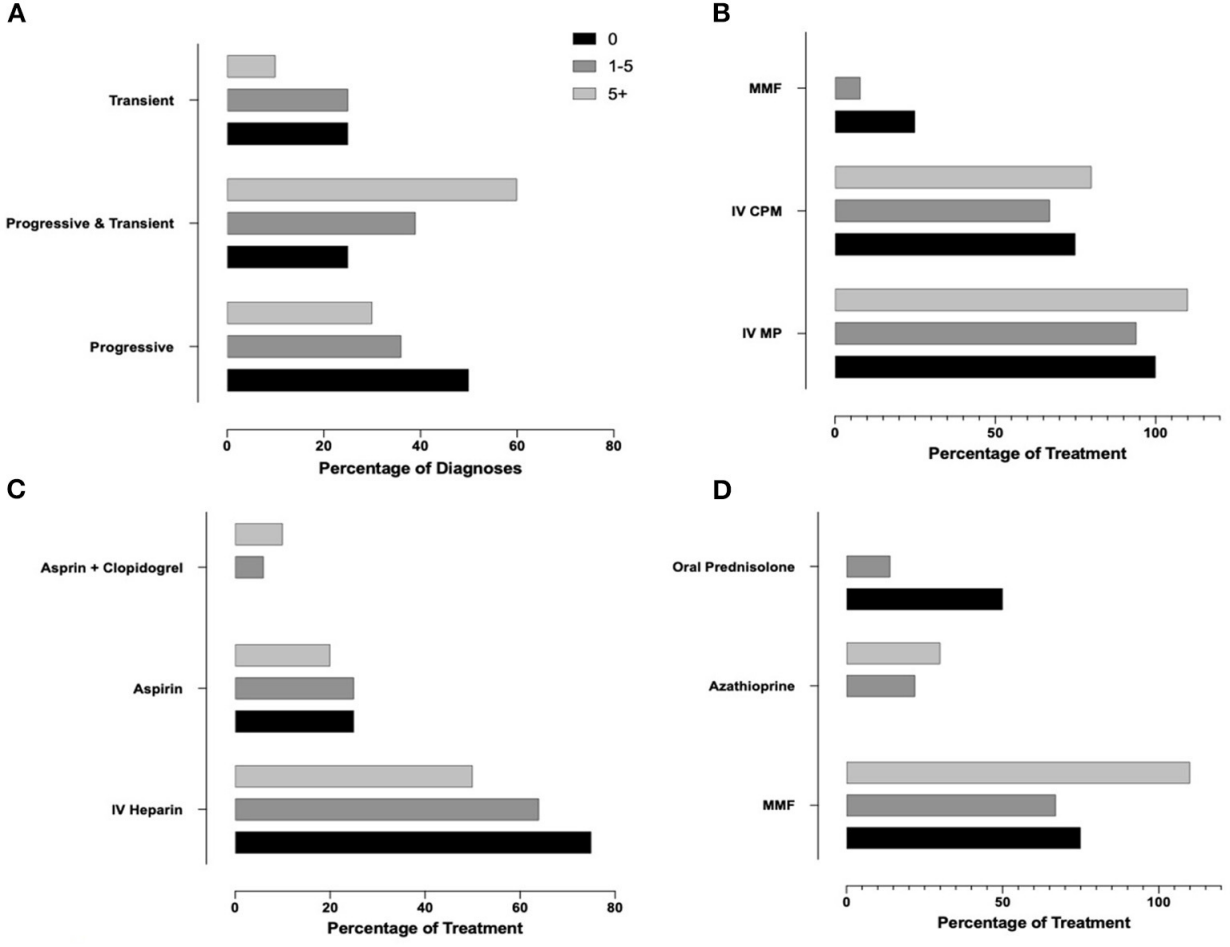
Influence of Experience in Approach to Diagnosis and Treatment in case 1. **(A)** Correlation between number of patients with p-cPACNS treated and their diagnosis of the patient in case 1. **(B)** Patients vs. Induction treatment. **(C)** Patients vs. Acute anticoagulation treatment. **(D)** Patients vs. Maintenance treatment.

**Figure 6:**
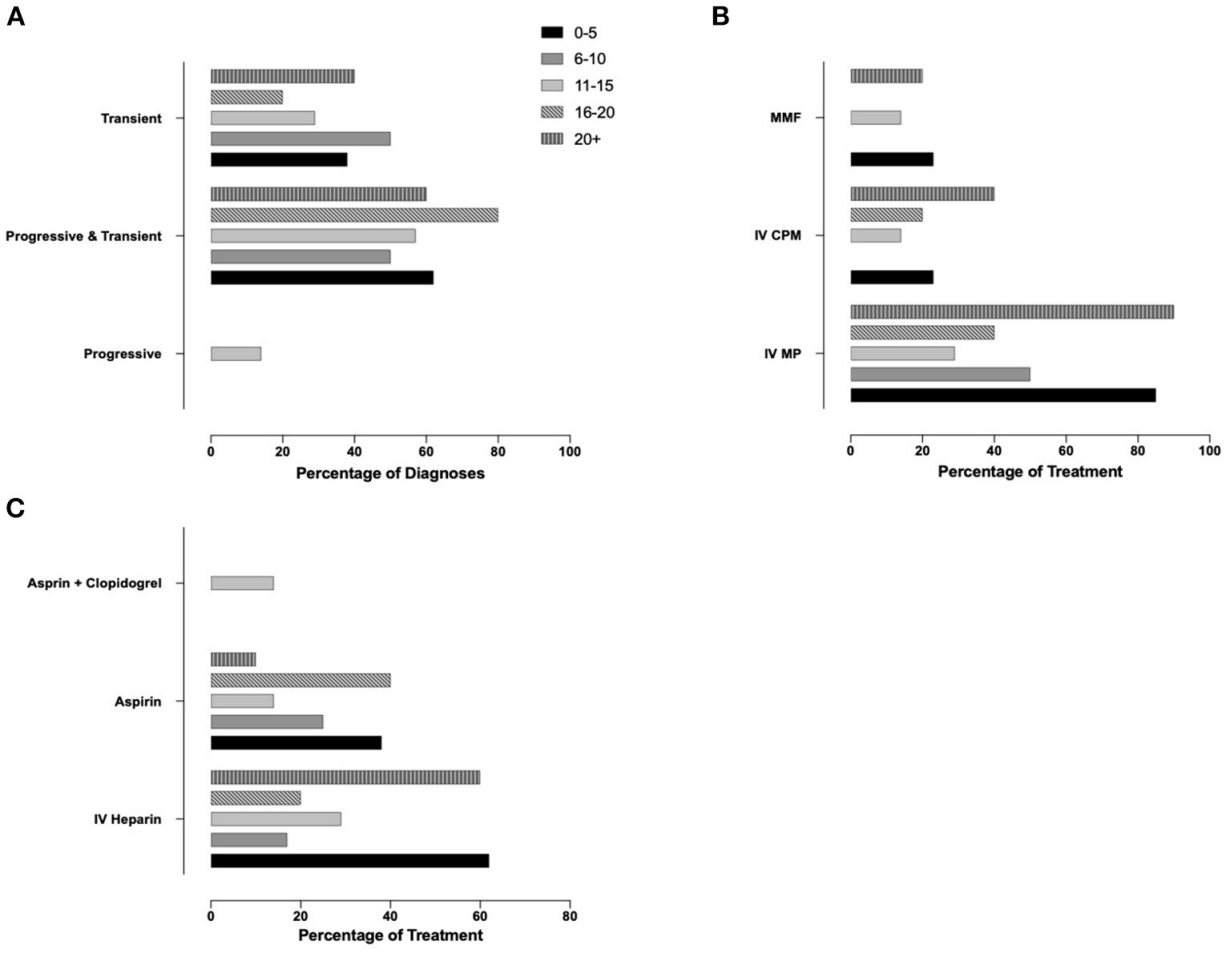
Influence of Experience in Approach to Diagnosis and Treatment in case 2. **(A)** Correlation between number of years of experience clinicians had and their diagnosis of the patient in case 2. **(B)** Years of experiences vs. Induction treatment. **(C)** Years of experience vs. Acute anticoagulation treatment.

**Figure 7:**
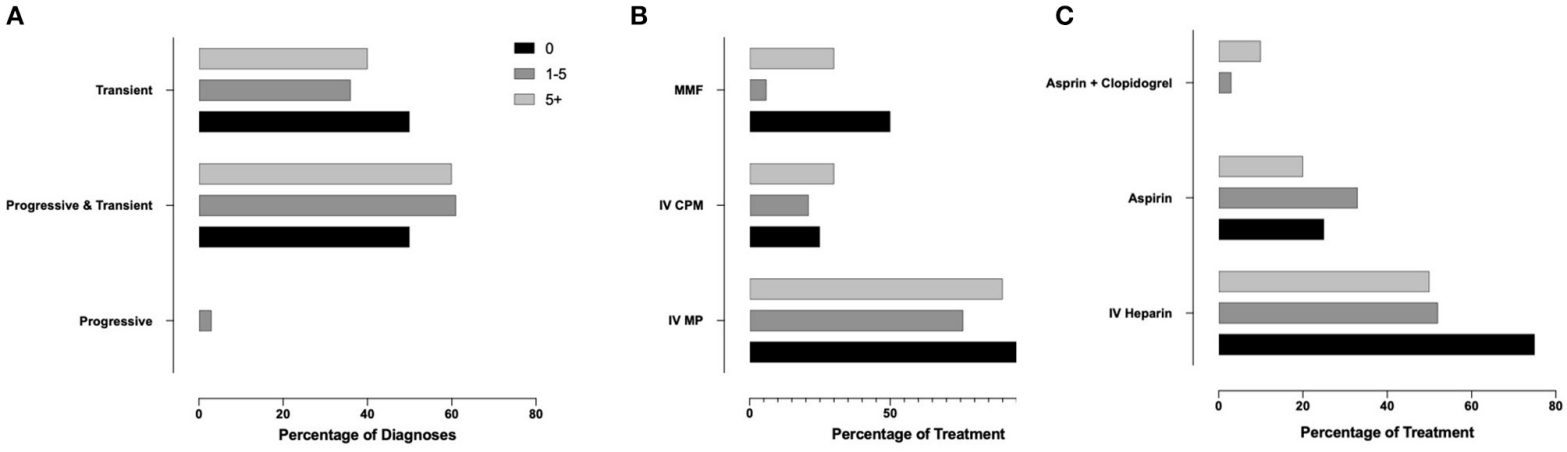
Influence of Experience in Approach to Diagnosis and Treatment in case 2. **(A)** Correlation between number of years of experience clinicians had and their diagnosis of the patient in case 2. **(B)** Years of experiences vs. Induction treatment. **(C)** Years of experience vs. Acute anticoagulation treatment.

Finally, we aimed to assess whether professional experience of the responders (years of practice, number of PACNS patients treated) associated with variable approaches to diagnosis and treatment (Q4, *N* = 91 responses). As responses varied significantly, especially among answers not within the top 3, only the top three most commonly selected answers for diagnosis and treatment methods were used. Responses related to case 1 were associated with years of experience (Supplementary Table 1, Figures 4A–D) and p-cPACNS patients treated (Supplementary Table 2, Figures 5A–D). In case 1, overall years of experience correlated with making the “correct diagnosis” likely progressive p-cPACNS (Figure 4A). Surprisingly, the number of patients treated (Figure 5A) did not correlate with making the “correct diagnosis” to the same extent. A similar trend was seen when considering the use of cyclophosphamide. Colleagues with more years of experience in their specialty less frequently considered cyclophosphamide, but more commonly MMF as induction treatment (Figure 4B), which also did not correlate as well with the number of cPACNS patients treated (Figure 5B). Response related to case 2 were also associated with years of experience (Supplementary Table 3, Figures 6A–C) and p-cPACNS patients treated (Supplementary Table 4, Figures 7A–C).

## Discussion

Childhood PACNS is a rare disease (13) that can cause significant damage, result in the death of individuals affected, or affect quality of life, psychomotor development, and lastly cause significant cost to healthcare systems (4). Diagnostic and therapeutic delay is common and caused by multiple factors, including a large number of differential diagnoses, overall lack of awareness, and the absence of standardized protocols for children with neurological deficits in many institutions. Furthermore, the absence of widely accepted, prospectively and independently evaluated diagnostic criteria and treatment algorithms, complicate the situation (14).

Especially in rare conditions it can be challenging to develop evidence-based and widely accepted diagnostic or classification criteria. The paucity of evidence for treatment is in part due to small patient numbers and therefore reduced interest by industry and funding bodies, and the fact that “standard-of-care” approaches are considered effective and the introduction of “placebo controls” would be unethical. Currently, diagnostic approaches and classification of cPACNS relies on suggested, but nor prospectively evaluated criteria, and treatment is based on mostly retrospective and relatively small case series and/or expert opinion (10). In such situations, establishing consensus on diagnostic and treatment plans to harmonize approaches and prospectively collect meaningful clinical datasets in relation to consensus treatment plans can be a helpful tool to generate evidence and allow improvement of outcomes in patients with rare diseases (11, 15–17).

The establishment of consensus treatment plans follows a structured approach, including surveying the current standard of care among experts as an early step (15–17). Here, we present results from an internationally shared online survey collecting information on diagnostic and therapeutic approaches of clinicians experienced with cPACNS.

Overall response rates were relatively low (ca. 1%), which may have been caused by limited experience with the condition across medical specialties and countries. Indeed, only 19.6% of responders had <5 years' clinical experience as a specialist suggesting that mostly more senior colleagues with personal experience in diagnosing and treating PACNS patients responded. Only 74.7% of participants had personal experience in the care of patients with cPACNS. This survey represents opinions from a relatively small number of pediatric rheumatologists, neurologists and other experts involved in the management of this rare condition. It will remain to be determined, e.g., through Delphi questionnaires and subsequent expert consensus meetings, whether responses are indeed widely representative for standard-of-care.

Diagnostic approaches for both forms of cPACNS queried here (progressive vs. transient p-cPACNS) did not vary between forms. Overall, 75.3% agreed that MRI is essential for diagnosing p-cPACNS, 26.8% favor lumbar puncture and CSF analyses, 20.2% would perform CT scans as an alternative to MRI. Interestingly, when confronted with the two case scenarios, responses were slightly different. MRI and blood laboratory tests were considered the most important and therefore likely most helpful tools with agreement between 90.5 and 94.3%, followed by CSF analyses (82.4 vs. 88.6%), and CT scans (23.0 vs. 31.4%).

Agreement regarding appropriate MRI strategies between respondents and cases was strong. Most participants would request MRI angiography (87.3 vs. 88.2%), followed by FLAIR (77.5 vs. 73.5%), diffusion weighted imaging (74.7 vs. 73.5%), T1 FS with contrast application (53.5 vs. 54.4), T2 FS (52.1 vs. 50.0%), T1 FS (42.3 vs. 41.2%), and lastly TIRM or STIR (39.4 vs. 39.7%). Based on published reports, MRI angiography is a strong tool to assess local perfusion and (to some extent) vessel obstruction (18), FLAIR sequences suppress cerebrospinal fluid (CSF) effects which is effective in identifying periventricular hyperintense lesions, such as multiple sclerosis plaques (19). Diffusion weighted imaging helps differentiating between new and old lesions and assess local perfusion in relation to vessel anomalies (20). As T1 sequences can be a strong tool to quantify vessel wall oedema and contrast enhancement can be a surrogate marker of active inflammation (21), it appears surprising that not more colleagues chose these examinations. However, radiologists were under-represented in this survey, but are certainly key members of multidisciplinary teams diagnosing and monitoring patients with cPACNS.

While patients with transient pPACNS can exhibit mild clinical symptoms indicative of focal neurological deficits, patients with progressive pPACNS usually present with symptoms of both focal and diffuse neurological deficits, including cognitive impairment, headaches and in some cases seizures (10, 22, 23). As 80.8% of participants agreed with the aforementioned approach to stratify patients by likely risk for disease progression and classified case 2 as transient p-cPACNS (81.4%), it appears surprising that 69.9% also stated that case 1 with posterior arteries affected may have transient disease.

Based on participants' responses, most important blood tests include blood cell counts and differentiation (99% both), clotting tests (97% both), immunology profiling (90–95%, including APL, ANA, ANCA, dsDNA, complement function, NMDA/aquaporin antibodies). Additional tests frequently requested included ADA2 levels for the exclusion of DADA2, and IGRA to exclude TBC, as both conditions have been reported to cause neuroinflammation/vasculitis (24, 25). Lumbar puncture/CSF analysis was suggested to include CSF opening pressure (85.6 vs. 84.3%), cell counts and protein (98.6 vs. 98.6%), microbiological cultures (93.2 vs. 90.0%), glucose levels (90.5 vs. 92.9%), oligoclonal bands (89.1 vs. 81.4%), lactate (74.3 vs. 78.5%), and NMDA/aquaporin antibodies (68.9 vs. 54.3%). Interestingly, as not included in the multiple-choice options and potentially helpful when considering infectious and/or reactive causes of vasculitis, none of the participants suggested virus serologies or PCRs in blood or CSF. However, virus diagnostics had been included in the previous question, which may have confused the participants.

Therapeutic approaches varied between participants and (not surprisingly) progressive vs. transient disease. In progressive p-cPACNS (case 1), most participants would have chosen an induction treatment regimen with IVMP (93.2%), followed by CPM (64.3%), MMF (12.3%) and oral prednisolone (4.1%), while in transient p-cPACNS (case 2), CPM was less frequently chosen (26.1%), while oral prednisolone appears to play a bigger role (13.0%). Provided potential side-effects and the transient nature of the disease, the use of CPM in transient p-cPACNS appears surprising, but prospective trials are lacking (3). Notably, evidence for a recent infection with VZV in transient p-cPACNS would significantly reduce the likelihood to prescribe CPM (2.9%) or MMF (2.9%). A majority of colleagues would include antiviral acyclovir in the treatment regimen (85.3%), and use of methylprednisolone was only considered by 47.1% (as compared to 76.8% without evidence of VZV). This appears somewhat surprising as vasculitis in VZV is likely of reactive nature and not caused by the virus itself (26), and patients may benefit from immune modulation. However, clinical evidence through trials does not exist. While overall agreement between less and more experienced colleagues was strong, years of experience associated with diagnostic certainty in case 1 (progressive p-cPACNS) and caution with the use of CPM, favoring MMF for induction treatment. This correlation was not seen when considering the number of PACNS patients treated. Indeed, more recently preliminary reports suggest that CPM may be replaced by alternatives, such as MMF in p-cPACNS (4) and other (systemic) autoimmune/inflammatory conditions, such as SLE (27). However, this may not apply to all individuals and has to be followed in prospective case collections/registers and clinical trials.

As maintenance treatment may only/mostly be necessary in cases with prolonged disease activity, it was only queried for progressive p-cPACNS. A majority of participants favor MMF (72.2%), followed by azathioprine (21%), prednisolone (18%), CPM (i.v., 14%, oral 1%), rituximab (10%), methotrexate (5%) and TNF inhibitors (1%). Responses reflect few available published reports in which aforementioned regimens had been chosen. However, data is limited to relatively small and retrospective case collections, and evidence from prospective trials is lacking.

Duration of corticosteroid treatment was recorded in progressive and transient p-cPACNS, and was largely comparable. Most colleagues would treat patients with transient disease for 3 (37.9%) or 6 months (34.9%), while slightly more would treat for 6 (41.7%) than 3 (20.8%) months in progressive p-cPACNS.

Antithrombotic prophylaxis was considered essential by a majority of participants in the acute and post-acute phase in both progressive and transient p-cPACNS. Heparin i.v., in the acute phase was deemed important in progressive (65.7%) and transient (52.2%) disease, followed by aspirin (27.1 vs. 29.9%), and the combination of aspirin and clopidogrel (7.1 vs. 6.0%). For post-acute phase prophylaxis, colleagues consider aspirin (47.1 vs. 53.0%), heparin s.c. (22.9 vs. 18.2%), aspirin and clopidogrel in combination (14.3 vs. 7.6%), clopidogrel (6 vs. 4.6%), DOACs (vs. 4.6% each). As many as 12.1% of colleagues would not prescribe post-acute phase thromboembolism prophylaxis in patients with transient p-cPACNS. Suggested duration of anticoagulant treatment did not vary dramatically, most colleagues suggested 12 months (18.6% in progressive and 20.6% in transient p-cPACNS), followed by 24 months (18.6 vs. 12.7%). Interestingly, few colleagues preferred 18 months treatment (4.3 vs. 3.2%). However, this reflects the absence of evidence and the urgent need for data collection and generation of consensus.

Proposed monitoring of disease activity and damage was comparable between both progressive and transient p-cPACNS, and, based on participants' responses, should include MRI imaging and clinical examinations every 3 months. However, agreement significantly reduced over time, and at 18 months only 15 vs. 12% found MRI and 26 vs. 23% clinical examinations to be of importance. This is special interest, as a majority of 73% would continue immune modulating treatment for up to 24 months, and flares appear more likely with or after treatment discontinuation.

Consensus on a multi-professional approach to diagnosis and treatment was strong for both transient and progressive p-cPACNS.

## Conclusion

In the absence of widely accepted and prospectively evaluated diagnostic criteria and evidence-based therapeutic strategies for p-cPACNS, clinical management varies between centers. Diagnostic and therapeutic strategies vary considerably, especially in regards to therapeutics used and treatment duration. Based on data from this survey, Delphi questionnaires will be developed to define statements to be used toward expert consensus meetings. This process will aim at the development of diagnostic and treatment plans for patients with p-cPACNS following agreed consensus-based protocols.

## Data Availability Statement

The original contributions presented in the study are included in the article/Supplementary Material, further inquiries can be directed to the corresponding author/s.

## Author Contributions

All authors were involved in study planning and manuscript preparation, AQ and CH lead on data analysis and preparation of the first written draft, all authors read and agreed on the final version of this manuscript.

## Conflict of Interest

The authors declare that the research was conducted in the absence of any commercial or financial relationships that could be construed as a potential conflict of interest.
