# Supplementary material for: Diagnosis and Treatment of Angiography Positive Medium to Large Vessel Childhood Primary Angiitis of Central Nervous System (p-cPACNS): An International Survey
Source: Front Pediatr. 2021 Mar 26;9:654537. doi: 10.3389/fped.2021.654537 (PMC8032958; doi:10.3389/fped.2021.654537)
Supplement: Supplementary file 1 [file Table_1.DOCX]

**Appendix**

**Supplement 1:**

**Medium/Large vessel CNS survey**

**Introductory Questions:**

1. What is your (sub-)specialty?
2. How many years’ experience have you had in this specialty?
3. What country are you currently working in?
4. Have you treated patients with large-vessel primary angiitis of the central nervous system (p-cPACNS)?

- If yes, how many?

**A clinical scenario will be outlined and you will be asked to answer questions on how you would systematically approach the patient and the treatment you would give.**

**Wie würden Sie bei folgenden klinischen Szenarien vorgehen?**

**Part 1**

1. **Please order the following exams following the order of importance for the diagnosis large vessel cPACNS in your opinion (1 being top choice, 7 being least important)**

- Blood inflammatory parameters, including CRP, ESR, blood cell counts (FBC/WCC)
- MRI Imaging: cerebral MRI, including angio-MRI
- Conventional Angiography
- Brain CT Scan, including CT Angio
- CSF Analysis: *cell number, protein, oligoclonal bands, lactate, glucose, opening pressure*
- CSF immunology *(encephalitis-associated antibodies)*
- Blood immunology (including ANA, ENA, including ds-DNA, ANCA, antiphospholipid antibodies, rheumatoid factor, complement system components and activation
- Others (please state):

1. **Do you believe genetics testing plays a role in diagnosing p-cPACNS (angiography positive childhood primary angiitis of the central nervous system)?**

- **If yes, please state which genes and/or genetic disorders you would investigate when diagnosing a patient with p-cPACNS**

**Part 2**

**Here, two clinical scenarios will be outlined and you will be asked answer questions regarding how you would systematically approach the patient and treatment you would give:**

**Case One**

**An 8-year-old boy presents with increasing fatigue (past two weeks) and new acute-onset symptoms of aphasia, ataxia, headaches, and progressive vertigo for the past 24 hours. He has no personal of family history of clotting disorders, strokes or autoimmune/inflammatory disease. He has had no infections in the past year and no travel history (other than a “sore throat or sniffles here and there”), no pets, and no other symptoms.**

1. **What first-line investigations would you perform? (Multiple answers possible)**

- Blood Tests (including full blood counts, inflammatory markers and clotting tests)
- Lumbar Puncture and CSF analysis, including cell counts and differentiation, protein, lactate, glucose, microbial cultures, herpes virus PCR, VZV PCR, Borrelia IgG, IgM
- Emergency MRI of the brain including angio-MRI
- Brain CT scan including CT angiography
- Others (please state)

1. **If you chose blood tests, which of the following bloods would you order?**

- N/A
- Full Blood Count (including complete white cell count)
- Clotting tests (including PTT, INR, fibrinogen, D dimers)
- Interferon Gamma Release Assay Test (for TB infection)
- Immunology (e.g. ANA, ENA, Complement Factors, Cardiolipin AB, ANCA)
- Adenosine deaminase 2 activity (ADA2)
- All of the above
- Would not perform bloods
- Others (please state)

1. **If you chose to look at blood immunology, which of the following would you look at?**

- N/A
- Antinuclear Antibodies (ANA)
- Anti-double stranded DNA (dsDNA)
- Complement factors and complement cascade activation
- Anti-phospholipid Antibodies
- Anti-neutrophil Cytoplasmic Antibody (ANCA)
- Anti-NMDA and aquaporin antibodies
- All of the above
- Would not look at blood immunology
- Others (please state)

1. **If you chose lumbar puncture, which of the following would you look at?**

| - N/A | - Culture |
| --- | --- |
| - LP opening pressure | - All of the above |
| - Cell count and differentiation | - Would not perform LP |
| - Protein | - Others (Please state) |
| - Lactate |  |
| - Oligoclonal Bands |  |
| - Anti-NMDA & aquaporin antibodies |  |
| - Glucose |  |

1. **If you chose Emergency MRI (within the same day), which of the following are you interested in?**

- N/A
- Diffusion-weighted MRI (DWI)
- T1 with fat saturation (FS)
- T1 FS, contract enhanced
- T2 FS
- TIRM (Turbo inversion recovery magnitude)/STIR (Short tau inversion recovery)
- FLAIR (Fluid-attenuated inversion recovery)
- MR Angiography
- All of the above
- Would not use MRI
- Other (please state)

**Patient exhibited elevated ESR (30mm/h) and CRP (4mg/L). Approximately 30h after the onset of ataxia and aphasia, Magnetic Resonance Imaging (MRI) shows alterations in proton diffusion capacity in the Cerebellum and a significant and long ranging stenosis of the distal Basilar artery in MRI angiography.**


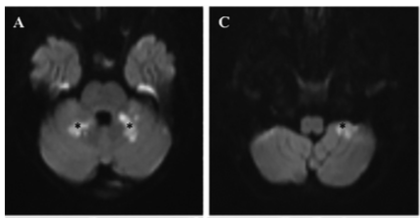

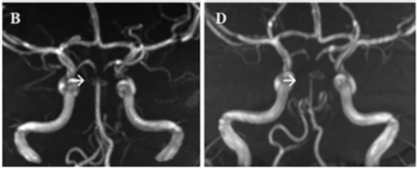


1. **What are your top differential diagnoses?**

- Migraine
- Infection, i.e. Meningitis
- Congenital deformity
- Medium sized vessel CNS vasculitis, likely transient
- Medium sized vessel CNS vasculitis, likely progressive
- Central nervous system Tuberculosis (CNS TB)
- AV Malformation
- Tumour
- Traumatic haemorrhage
- Ischemic stroke
- Multiple Sclerosis
- Others (please state):

1. **Which (early) criteria would you use to make the diagnosis (likely) progressive p-cPACNS? /**

- Clinical course (progression after 3 months)
- Clinical acute presentation with “systemic signs”
- Laboratory Findlings (Systemic Inflammation)
- Imaging (more than one vessel affected, distal segments affected, posterior vessels affected)
- Response to immune modulation
- Other criteria (which ones?)

**Autoantibodies in CSF and blood come back negative, there’s no evidence for clotting disorders or infection, including TB. Systemic inflammatory parameters remain normal. Based on the involvement of posterior arteries, the diagnosis of (likely) progressive p-cPACNS (Childhood Primary Angiitis of Central Nervous System) is made. What medication would you start the patient on?**

1. **What medication would you give the patient?**

- Initiation with IV Methylprednisolone (IVMP) over 5 days (20-30mg/kg/day, up to 1000mg), followed by oral Prednisolone staring at 2 mg/kg/day, up to 100mg/day
- Initiation with oral Prednisolone (2 mg/kg/day, up to 100mg/day), followed by oral Prednisolone taper
- Intravenous Cyclophosphamide (500-750mg/m^2^ i.v. every 4 weeks for 4-6 months)
- Oral Cyclophosphamide following Fauci-Scheme
- Mycophenolate Mofetil (MMF) induction treatment (900-1200 mg/m^2^/day)
- Azathioprine (1.5-2.5mg/kg/day)
- Other (please state)

1. **Which acute anticoagulation treatment would you consider?**

- Initially Heparin i.v. (100-150 units/kg/day)
- Aspirin
- Warfarin
- Clopidogrel
- Combination of Aspirin and Clopidogrel
- Direct Oral Anticoagulants (DOACs) e.g. Apixaban, Rivaroxaban, Betrixaban
- Other (please state)

1. **Which post-acute anticoagulation treatment would you consider?**

- Heparin s.c.
- Aspirin
- Warfarin
- Clopidogrel
- Combination of Aspirin and Clopidogrel
- Direct Oral Anticoagulants (DOACs) e.g. Apixaban, Rivaroxaban, Betrixaban
- Other (please state)

1. **Which immune modulating maintenance treatment would you consider?**

- Cyclophosphamide i.v.
- Cyclophosphamide following Fauci scheme
- Mycophenolate Mofetil (MMF: 900-1200 mg/m^2^/day)
- Methotrexate (10-20mg/m^2^/week)
- Oral prednisolone
- Azathioprine (1.5-2.5mg/kg/day)
- Rituximab (375mg/m^2^ four times, repeat as needed)
- Anti-TNF agent (Infliximab, Adalimumab, etc.)
- Would not administer immune modulating maintenance treatment
- Other (please state)

1. **How long would immune modulating maintenance treatment be required for, in your opinion?**

| - 3 months | - 24 months |
| --- | --- |
| - 6 months | - 36 months |
| - 12 months | - Other (Please state) |
| - 18 months | - Would not administer treatment |

1. **How long would you give oral corticosteroids treatment for (including slow taper)?**

| - 3 months | - 24 months |
| --- | --- |
| - 6 months | - 36 months |
| - 12 months | - Other (Please state) |
| - 18 months | - Would not administer treatment |

1. **When would you discontinue anticoagulation treatment?**

| - 3 months | - 24 months |
| --- | --- |
| - 6 months | - 36 months |
| - 12 months | - Other (Please state) |
| - 18 months | - Would not administer treatment |

1. **When would you repeat MRI?**

| - 3 months | - 24 months |
| --- | --- |
| - 6 months | - 36 months |
| - 12 months | - Other (Please state) |
| - 18 months |  |

1. **When would you want a clinical follow up?**

| - 3 months | - 24 months |
| --- | --- |
| - 6 months | - 36 months |
| - 12 months | - Other (Please state) |
| - 18 months |  |

1. **What Specialities do you believe should be involved in the treatment of Case 1? (Multiple Options)**

| - Pediatric Neurology | - Oncology |
| --- | --- |
| - Rheumatology | - Intensive care |
| - Hematology | - Infectious Diseases |
| - Radiology | - All of the above |

**Case Two**

**A 4-year-old girl presents with headaches and symptoms suggestive of a cerebrovascular stroke (vomiting with some language and speech delays). She has a past medical history of a clinically diagnosed Varicella Zoster Virus (VZV) infection 6 months ago. There’s no history of strokes or clotting disorders in her personal or family history.**

**Part 2A**

1. **What first-line investigations would you perform?**

- Blood Tests (including full blood counts, inflammatory markers and clotting tests)
- Lumbar Puncture and CSF analysis, including cell counts and differentiation, protein, lactate, glucose, microbial cultures, herpes virus PCR, VZV PCR, Borrelia IgG, IgM
- Emergency MRI of the brain including angio-MRI
- Brain CT scan including CT angiography
- Others (please state)

1. **If you chose blood tests, which of the following bloods would you order?**

- N/A
- Full Blood Count (including complete white cell count)
- Clotting tests (including PTT, INR, fibrinogen, D dimers)
- Interferon Gamma Release Assay Test (for TB infection)
- Immunology (e.g. ANA, ENA, Complement Factors, Cardiolipin AB, ANCA)
- Adenosine deaminase 2 activity (ADA2)
- All of the above
- Would not perform blood tests
- Others (please state)

1. **If you chose to look at blood immunology, which of the following would you look at?**

- N/A
- Antinuclear Antibodies (ANA)
- Anti-double stranded DNA (dsDNA)
- Complement factors and complement cascade activation
- Anti-phospholipid Antibodies
- Anti-neutrophil Cytoplasmic Antibody (ANCA)
- Anti-NMDA and aquaporin antibodies
- All of the above
- Would not look at blood immunology
- Others (please state)

1. **If you chose lumbar puncture, which of the following would you look at?**

- N/A
- LP opening pressure
- Cell count and differentiation
- Protein
- Lactate
- Oligoclonal Bands
- Anti-NMDA and aquaporin antibodies
- Glucose
- Culture
- All of the above
- Would not perform a lumbar puncture
- Others (please state)

1. **If you chose Emergency MRI, which of the following are you interested in?**

- N/A
- Diffusion-weighted MRI (DWI)
- T1 with fat saturation (FS)
- T1 FS, contract enhanced
- T2 FS
- TIRM (Turbo inversion recovery magnitude)/STIR (Short tau inversion recovery)
- FLAIR (Fluid-attenuated inversion recovery)
- MR Angiography
- All of the above
- Would not want an MRI
- Other (please state)

**Autoantibodies in CSF and blood come back negative, there’s no evidence for clotting disorders or infection (including negative for TB and VZV PCR in CSF, serum VZV IgG positive, IgM borderline positive). Blood and CSF inflammatory markers remain within normal limits.**

**DWI sequences unveiled altered diffusion capacity in the left hemisphere; Time of flight MR Angiography (TOF-MRA-)sequences demonstrate narrow caliber of left distal internal carotid artery and proximal anterior and medial cerebral artery. Post-gadolinium MRI sequences reveal contrast enhancement of the thickened vascular wall in the affected segments. Conventional angiography showed incomplete occlusion of the left A. cerebri media.**

^
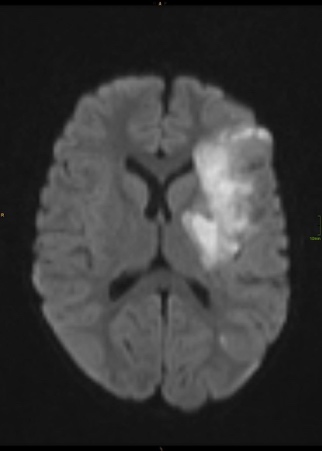
^
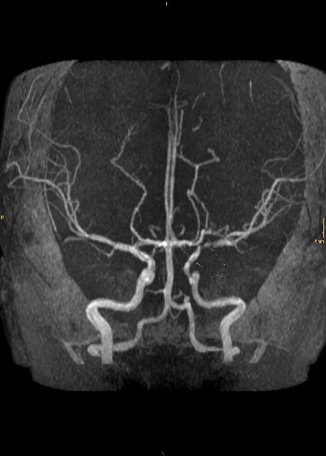

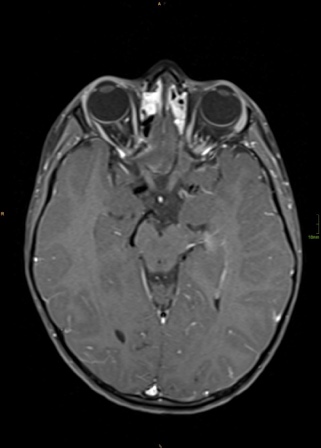

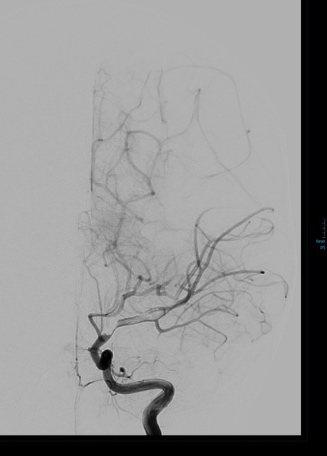


1. **Based on the patient’s history, what are the most important differentials to consider? Chose top 5**

- Migraine
- Infection, i.e. Meningitis
- Congenital deformity
- Medium sized vessel CNS vasculitis, likely transient related to VZV
- Medium sized vessel CNS vasculitis, likely transient not related to VZV
- Medium sized vessel CNS vasculitis, likely progressive
- Central nervous system Tuberculosis (CNS TB)
- Tumour
- Traumatic haemorrhage
- Ischemic stroke
- Multiple Sclerosis
- Others (please state: ________)

1. **What medication would you give the patient (multiple choices possible)?**

- Initiation with IVMP over 5 days (20-30mg/kg/day, up to 1000mg), followed by oral Prednisolone staring at 2 mg/kg/day, up to 100mg/day
- Oral Prednisolone (2 mg/kg/day, up to 100mg/day), followed by oral Pred taper
- Intravenous Cyclophosphamide (500-750mg/m^2^ i.v. every 4 weeks for 4-6 months)
- Oral Cyclophosphamide following Fauci-Scheme
- Mycophenolate Mofetil (MMF) induction treatment (900-1200 mg/m^2^/day)
- Azathioprine (1.5-2.5mg/kg/day)
- Acyclovir treatment i.v. over 14 days
- Other (please state)

1. **Which acute anticoagulation treatment would you consider?**

- Initially Heparin i.v. (100-150 units/kg/day)
- Aspirin
- Warfarin
- Clopidogrel
- Combination of Aspirin and Clopidogrel
- Direct Oral Anticoagulants (DOACs) e.g. Apixaban, Rivaroxaban, Betrixaban
- Would not administer anticoagulation treatment
- Other (please state)

1. **Which post-acute anticoagulation treatment would you consider?**

- Heparin s.c.
- Aspirin
- Warfarin
- Clopidogrel
- Combination of Aspirin and Clopidogrel
- Direct Oral Anticoagulants (DOACs) e.g. Apixaban, Rivaroxaban, Betrixaban
- Would not administer post-acute anticoagulation treatment
- Other (please state)

1. **How long would immune modulating maintenance treatment be required for, in your opinion?**

| - 3 months | - 24 months |
| --- | --- |
| - 6 months | - 36 months |
| - 12 months | - Other (Please state) |
| - 18 months | - Would not administer treatment |

1. **How long would you give oral corticosteroids treatment for (including slow taper)?**

| - 3 months | - 24 months |
| --- | --- |
| - 6 months | - 36 months |
| - 12 months | - Other (Please state) |
| - 18 months | - Would not administer treatment |

1. **When would you discontinue anticoagulation treatment?**

| - 3 months | - 24 months |
| --- | --- |
| - 6 months | - 36 months |
| - 12 months | - Other (Please state) |
| - 18 months | - Would not administer treatment |

1. **When would you repeat MRI? (Multiple options)**

| - 3 months | - 24 months |
| --- | --- |
| - 6 months | - 36 months |
| - 12 months | - Other (Please state) |
| - 18 months | - Would not perform repeat MRI |

1. **When would you want a Clinical follow up? (Multiple options)**

| - 3 months | - 24 months |
| --- | --- |
| - 6 months | - 36 months |
| - 12 months | - Other (Please state) |
| - 18 months | - Would not want a repeat clinical follow up |

**Part 2B**

**After the first line investigations, suppose the autoantibodies in CSF and blood come back negative, there’s no evidence for clotting disorders, but the VZV PCR in the CSF comes back as positive.**

1. **What medication would you give the patient (multiple choices possible)?**

- Initiation with IV Methylprednisolone (IVMP) over 5 days (20-30mg/kg/day, up to 1000mg), followed by oral Prednisolone staring at 2 mg/kg/day, up to 100mg/day
- Initiation with oral Prednisolone (2 mg/kg/day, up to 100mg/day), followed by oral Prednisolone taper
- Intravenous Cyclophosphamide (500-750mg/m^2^ i.v. every 4 weeks for 4-6 months)
- Oral Cyclophosphamide following Fauci-Scheme
- Mycophenolate Mofetil (MMF) induction treatment (900-1200 mg/m^2^/day)
- Azathioprine (1.5-2.5mg/kg/day)
- Acyclovir treatment i.v. over 14 days
- Other (please state)

1. **What Specialities do you believe should be involved in the treatment of Case 2? (Multiple Options)**

- Paediatric Neurology
- Rheumatology
- Haematology
- Radiology
- Oncology
- Intensive Care
- Infectious Diseases
- All of the Above
- Other (please state)
