# Supplementary material for: Diagnosis and Treatment of Angiography Positive Medium to Large Vessel Childhood Primary Angiitis of Central Nervous System (p-cPACNS): An International Survey
Source: Front Pediatr. 2021 Mar 26;9:654537. doi: 10.3389/fped.2021.654537 (PMC8032958; doi:10.3389/fped.2021.654537)
Supplement: Supplementary file 2 [file Table_2.docx]

***Supplement Table 1:* Correlated data to determine if clinicians with more years of experience diagnose and treat PACNS patients differently using the information provided in case 1.**

| **Years of experience** | **Diagnosis (%)** | | | **Treatment (%)** | | | | | | | | |
| --- | --- | --- | --- | --- | --- | --- | --- | --- | --- | --- | --- | --- |
|  |  |  |  | **Induction** | | | **Acute Anticoagulant** | | | **Maintenance** | | |
|  | **Progressive PACNS** | **Progressive & Transient PACNS** | **Transient PACNS** | **IV Methylprednisolone** | **IV Cyclophosphamide** | **MMF** | **IV Heparin** | **Aspirin** | **Aspirin & Clopidogrel** | **MMF** | **Azathioprine** | **Oral Prednisolone** |
| **0-5 (n=13)** | 46.2 | 46.2 | 7.7 | 92.3 | 76.9 | 7.7 | 76.9 | 30.8 | 0.0 | 69.2 | 15.4 | 23.1 |
| **6-10 (n=17)** | 17.6 | 52.9 | 23.5 | 94.1 | 47.1 | 5.9 | 52.9 | 35.3 | 0.0 | 64.7 | 23.5 | 17.6 |
| **11-15 (n=14)** | 28.6 | 42.9 | 21.4 | 100.0 | 71.4 | 14.3 | 57.1 | 14.3 | 7.1 | 85.7 | 21.4 | 7.1 |
| **16-20 (n=8)** | 87.5 | 12.5 | 0 | 100.0 | 87.5 | 12.5 | 37.5 | 50.0 | 12.5 | 75.0 | 50.0 | 12.5 |
| **20+ (n=21)** | 23.8 | 57.1 | 4.8 | 95.2 | 57.1 | 19.0 | 71.4 | 14.3 | 14.3 | 76.2 | 14.3 | 19.0 |

Q2, 12, 14, 15 and 17 was used to determine if there was a link between the number of years of experience respondents had to their diagnosis and treatment method. Only the three most popular answers were used from each category.

***Supplement Table 2:* Correlated data to determine if clinicians who have treated more PACNS patients diagnose and treat these patients differently using the information provided in case 1.**

| **Number of PACNS patients treated** | **Diagnosis (%)** | | | **Treatment (%)** | | | | | | | | |
| --- | --- | --- | --- | --- | --- | --- | --- | --- | --- | --- | --- | --- |
|  |  |  |  | **Induction** | | | **Acute Anticoagulant** | | | **Maintenance** | | |
|  | **Progressive PACNS** | **Progressive & Transient PACNS** | **Transient PACNS** | **IV Methylprednisolone** | **IV Cyclophosphamide** | **MMF** | **IV Heparin** | **Aspirin** | **Aspirin & Clopidogrel** | **MMF** | **Azathioprine** | **Oral Prednisolone** |
| **0 (n=4)** | 50.0 | 25.0 | 25.0 | 100.0 | 75.0 | 25.0 | 75.0 | 25.0 | 0.0 | 75.0 | 0.0 | 50.0 |
| **1-5 (n=36)** | 38.9 | 38.9 | 16.7 | 94.4 | 66.7 | 8.3 | 63.9 | 25.0 | 5.6 | 66.7 | 22.2 | 13.9 |
| **5+ (n=10)** | 27.3 | 54.5 | 9.1 | 100.0 | 70.0 | 0.0 | 50.0 | 20.0 | 10.0 | 100.0 | 30.0 | 0.0 |

Q4, 12, 14, 15 and 17 was used to determine if there was a link between the number of PACNS patients the clinicians had treated to their diagnosis and treatment methods. Only the three most popular answers were used from each category.

***Supplement Table 3:* Correlated data to determine if clinicians with more years of experience diagnose and treat PACNS patients differently using the information provided in case 2.**

| **Years of experience** | **Diagnosis (%)** | | | **Treatment (%)** | | | | | | |
| --- | --- | --- | --- | --- | --- | --- | --- | --- | --- | --- |
|  |  |  |  | **Induction** | | | **Acute Anticoagulant** | | | |
|  | **Progressive PACNS** | **Progressive & Transient PACNS** | **Transient PACNS** | **IV Methylprednisolone** | **IV Cyclophosphamide** | **MMF** | **IV Heparin** | **Aspirin** | **Aspirin & Clopidogrel** |  |
| **0-5 (n=13)** | 0.0 | 61.5 | 38.5 | 84.6 | 23.1 | 23.1 | 61.5 | 38.5 | 0.0 |  |
| **6-10 (n=16)** | 0.0 | 50.0 | 50.0 | 81.3 | 6.3 | 6.3 | 50.0 | 37.5 | 0.0 |  |
| **11-15 (n=14)** | 14.3 | 64.3 | 21.4 | 64.3 | 21.4 | 21.4 | 35.7 | 28.6 | 14.3 |  |
| **16-20 (n=7)** | 0.0 | 71.4 | 28.6 | 35.7 | 21.4 | 14.3 | 28.6 | 21.4 | 7.1 |  |
| **20+ (n=21)** | 0.0 | 57.1 | 38.1 | 95.2 | 38.1 | 19.0 | 61.9 | 14.3 | 4.8 |  |

Q2, 29, 30 and 31 was used to determine if there was a link between the number of years of experience respondents had to their diagnosis and treatment method. Only the three most popular answers were used from each category.

**Supplement Table 4: Correlated data to determine if clinicians who have treated more PACNS patients diagnose and treat these patients differently using the information provided in case 2.**

| **Number of PACNS patients treated** | **Diagnosis (%)** | | | **Treatment (%)** | | | | | |
| --- | --- | --- | --- | --- | --- | --- | --- | --- | --- |
|  |  |  |  | **Induction** | | | **Acute Anticoagulant** | | |
|  | **Progressive PACNS** | **Progressive & Transient PACNS** | **Transient PACNS** | **IV Methylprednisolone** | **IV Cyclophosphamide** | **MMF** | **IV Heparin** | **Aspirin** | **Aspirin & Clopidogrel** |
| **0 (n=4)** | 0.0 | 50.0 | 50.0 | 100.0 | 25.0 | 50.0 | 75.0 | 25.0 | 0.0 |
| **1-5 (n=34)** | 2.9 | 61.8 | 35.3 | 76.5 | 20.6 | 8.8 | 50.0 | 32.4 | 2.9 |
| **5+ (n=10)** | 0.0 | 60.0 | 40.0 | 90.0 | 30.0 | 30.0 | 50.0 | 20.0 | 10.0 |

Q4, 29, 30 and 31 was used to determine if there was a link between the number of PACNS patients the clinicians had treated to their diagnosis and treatment methods. Only the three most popular answers were used from each category.
